# The association between newborn cord blood steroids and ambient prenatal exposure to air pollution: findings from the ENVIR*ON*AGE birth cohort

**DOI:** 10.1186/s12940-023-01010-w

**Published:** 2023-09-07

**Authors:** Michelle Plusquin, Congrong Wang, Charlotte Cosemans, Harry A. Roels, Maartje Vangeneugden, Bruno Lapauw, Tom Fiers, Guy T’Sjoen, Tim S. Nawrot

**Affiliations:** 1 Centre for Environmental Sciences, UHasselt, Diepenbeek, Belgium; 2 Department of Endocrinology, Ghent University Hospital, Ghent, Belgium; 3 Department of Clinical Pathology, Ghent University Hospital, Ghent, Belgium; 4 Department of Public Health & Primary Care, Leuven University, Leuven, Belgium

**Keywords:** Steroid, Cord blood, Air pollution, Particulate matter, Black carbon, ENVIR*ON*AGE, BKMR-DLM

## Abstract

Knowledge of whether prenatal exposure to ambient air pollution disrupts steroidogenesis is currently lacking. We investigated the association between prenatal ambient air pollution and highly accurate measurements of cord blood steroid hormones from the androgenic pathway.

This study included 397 newborns born between the years 2010 and 2015 from the ENVIR*ON*AGE cohort in Belgium of whom six cord blood steroid levels were measured: 17α-hydroxypregnenolone, 17α-hydroxyprogesterone, dehydroepiandrosterone, pregnenolone, androstenedione, and testosterone. Maternal ambient exposure to PM_2.5_ (particles with aerodynamic diameter ≤ 2.5 μm), NO_2,_ and black carbon (BC) were estimated daily during the entire pregnancy using a high-resolution spatiotemporal model. The associations between the cord blood steroids and the air pollutants were tested and estimated by first fitting linear regression models and followed by fitting weekly prenatal exposures to distributed lag models (DLM). These analyses accounted for possible confounders, coexposures, and an interaction effect between sex and the exposure. We examined mixture effects and critical exposure windows of PM_2.5_, NO_2_ and BC on cord blood steroids via the Bayesian kernel machine regression distributed lag model (BKMR-DLM).

An interquartile range (IQR) increment of 7.96 µg/m^3^ in PM_2.5_ exposure during pregnancy trimester 3 was associated with an increase of 23.01% (99% confidence interval: 3.26–46.54%) in cord blood levels of 17α-hydroxypregnenolone, and an IQR increment of 0.58 µg/m³ in BC exposure during trimester 1 was associated with a decrease of 11.00% (99% CI: -19.86 to -0.012%) in cord blood levels of androstenedione. For these two models, the DLM statistics identified sensitive gestational time windows for cord blood steroids and ambient air pollution exposures, in particular for 17α-hydroxypregnenolone and PM_2.5_ exposure during trimester 3 (weeks 28–36) and for androsterone and BC exposure during early pregnancy (weeks 2–13) as well as during mid-pregnancy (weeks 18–26). We identified interaction effects between pollutants, which has been suggested especially for NO_2_.

Our results suggest that prenatal exposure to ambient air pollutants during pregnancy interferes with steroid levels in cord blood. Further studies should investigate potential early-life action mechanisms and possible later-in-life adverse effects of hormonal disturbances due to air pollution exposure.

**Supplementary Information:**

The online version contains supplementary material available at 10.1186/s12940-023-01010-w.

## Highlights

PM_2.5_ exposure during trimester 3 is positively associated with cord blood 17α-hydroxypregnenolone.

Black carbon exposure during pregnancy is negatively associated with cord blood androstenedione.

Air pollutants during pregnancy interfere with *in utero* steroid levels in cord blood.

**Supplementary Information:**

The online version contains supplementary material available at 10.1186/s12940-023-01010-w.

## Introduction

Steroid hormones are involved in many biological processes during *in utero* development such as reproductive and metabolic programming, and neuroendocrine mechanisms [1]. The sex steroids in human blood include androgens [testosterone, dehydro-epiandrosterone (DHEA), dehydro-epiandrosterone sulfate (DHEAS), androstenedione, and dihydro-testosterone], estrogens (estradiol, estriol, and estrone), and progestogens (progesterone and 17α-hydroxyprogesterone) [2]. Steroids are important for regulating a multitude of functions throughout gestation, including fetal maturation and growth contributing significantly to the progression and outcome of the pregnancy [3]. The placenta has a central role in the biosynthesis of steroids throughout gestation, as it serves as the primary organ responsible for this process. Placental steroid biosynthesis requires maternal low-density lipoproteins, which are transferred to the placental syncytiotrophoblast where cytochrome P450 enzymes hydroxylate and cut the cholesterol side chain, producing pregnenolone. Based on this molecule the placental syncytiotrophoblast layer primarily secretes progesterone and estrogens, whereas fetal organs are a source of androgens and corticosteroids [4]. During pregnancy the fetal steroid profile is influenced by various sources, including maternal production, placental production, and the transfer of hormones across the placenta which in turn has an influence on the hormone levels in cord blood [5].

Compounds such as industrial chemicals, combustion byproducts, pesticides, herbicides, and metals can be present in the air as volatile or semi-volatile compounds, either in a gaseous state or attached to particulate matter [6]. Among these compounds are endocrine-disrupting compounds (EDCs) [6], chemical substances that may cause adverse health effects to the intact human organism and/or its progeny following changes in endocrine function [7]. These pollutants or airborne particles have the potential to disrupt the regular functioning of the endocrine system via endocrine disruption or by inducing systemic inflammation [6]. These disruptions can also affect the reproductive system, potentially leading to various reproductive health issues. For example, in adult men, higher ambient air pollution exposure has been significantly associated with a lower serum sex hormones level [8, 9] and evidence suggests a link between ambient air pollution and sperm quality [10]. While in pregnant women, a recent study showed that exposure to ambient fine particulate matter during the pre-conception and early prenatal stages could result in changes to steroid adaptation during pregnancy, potentially impacting the health of both the mother and child [11].

A few epidemiological studies found a link between ambient air pollution exposure and delayed pubertal onset, a process highly dependent on the functioning of sex hormones [12–14]. About 20 years ago, a study among adolescents living in the surroundings of a waste incinerator in Antwerp (Flanders, Belgium) found serum levels of exposure to polychlorinated biphenyls and dioxin-like compounds that suggested interference of these exposures with the adolescents’ sexual maturation [15]. Another study investigated pubertal levels of testosterone and estradiol in relation to air pollution but did not find evidence of an association [16].

Early-life events may play a role in adulthood by influencing susceptibility later in life to chronic diseases [17]. As such, *in utero* development occurs during a time window with particular sensitivity to toxic exposures. Prenatal exposure to ambient air pollutants has been associated with several adverse health outcomes at birth and/or childhood, such as an increased risk of low birth weight [18, 19], prematurity [20], neurodevelopmental alterations [21], and cancers [22]. Epidemiological studies found sex-specific effects of air pollution on neurodevelopmental outcomes [21, 23], which have been corroborated by animal studies suggesting sex differences in the association between prenatal air pollution and neurodevelopment in the offspring [24–26].

Knowledge on whether exposure to air pollution disrupts *in utero* steroid levels is currently lacking. To address this gap, we investigated the association between ambient air pollution exposure of PM_2.5_ (particles with aerodynamic diameter ≤ 2.5 μm), NO_2_ (nitrogen dioxide), and BC (black carbon)] at the maternal home residence(s) during pregnancy as a proxy for the prenatal exposure to ambient air pollution and the levels of highly sensitive hormone measurements in cord blood including steroids from the male hormonal pathway (17α-hydroxypregnenolone, 17α-hydroxyprogesterone, dehydroepiandrosterone, pregnenolone, androstenedione, and testosterone). In addition, we identified susceptible gestational time windows of air pollution exposure on cord blood levels of steroids.

## Methods

### Participants

For the current study, we included 400 mother-newborn pairs from the prospective birth cohort study ENVIR*ON*AGE (ENVIRONmental influence *ON* AGEing in early life) [27]. The study has been approved by the ethical committees of Hasselt University and the East-Limburg Hospital and was conducted according to the ethical principles in the Helsinki Declaration. Mother-newborn pairs are recruited upon their arrival for delivery at the East-Limburg Hospital in Genk (Belgium). Mothers without a planned cesarean section and able to fill out a Dutch language questionnaire are eligible for the cohort, and all participating mothers provided written informed consent. The catchment area of the hospital includes the province of Limburg, (Flanders, Belgium) which combines urban, suburban and rural areas. Recruitment started in February 2010 and is currently still ongoing.

The mothers completed the study questionnaires in the post-delivery ward to provide detailed information on gestational age (weeks), pre-pregnancy body mass index (BMI, kg/m^2^), maternal education (“low”, no diploma or primary school; “middle”, high school; or “high”, college or university degree), continued smoking during pregnancy (yes/no), alcohol consumption (yes/no), maternal age (years), maternal weight gain during pregnancy (kg), the season of delivery (winter, spring, summer, autumn), birth weight (g), parity (1 child, 2 or more children), medication use during the pregnancy (yes/no), caesarian section (yes/no), exposed to second-hand smoke indoor (yes, no), and the newborn’s ethnicity. Based on the native country of the newborn’s grandparents, we classified his/her ethnicity as European-Caucasian when two or more grandparents were European or non-European when at least three grandparents were of non-European origin. Gestational age was based on the date of conception, estimated based on maternity records of the last menstrual period, and if not available based on the first ultrasound exam. The biological sex was collected from questionnaires and verified in the medical records.

The neonate-mother pairs of this study were included between April 2010 and June 2015, included only singletons and were randomly selected based on the availability of core variables. Due to missing exposure measurements (*n* = 3) the sample size of the study population for statistical analysis was n = 397 for all exposures except for black carbon (n missing = 24) for which the sample size was n = 378.

### Biosamples

Cord blood (mixture of arterial and venous blood) was collected directly after delivery in BD Vacutainer® plastic tubes with spray-coated K2EDTA (BD, Franklin Lakes, NJ, USA). Within 20 min of blood collection, the tubes were centrifuged at 3,200 rpm for 15 min to separate the plasma that was stored in Eppendorf® tubes at − 80 °C until steroid hormone analysis.

### Steroid hormone analyses in cord blood

**Fig. 1:**
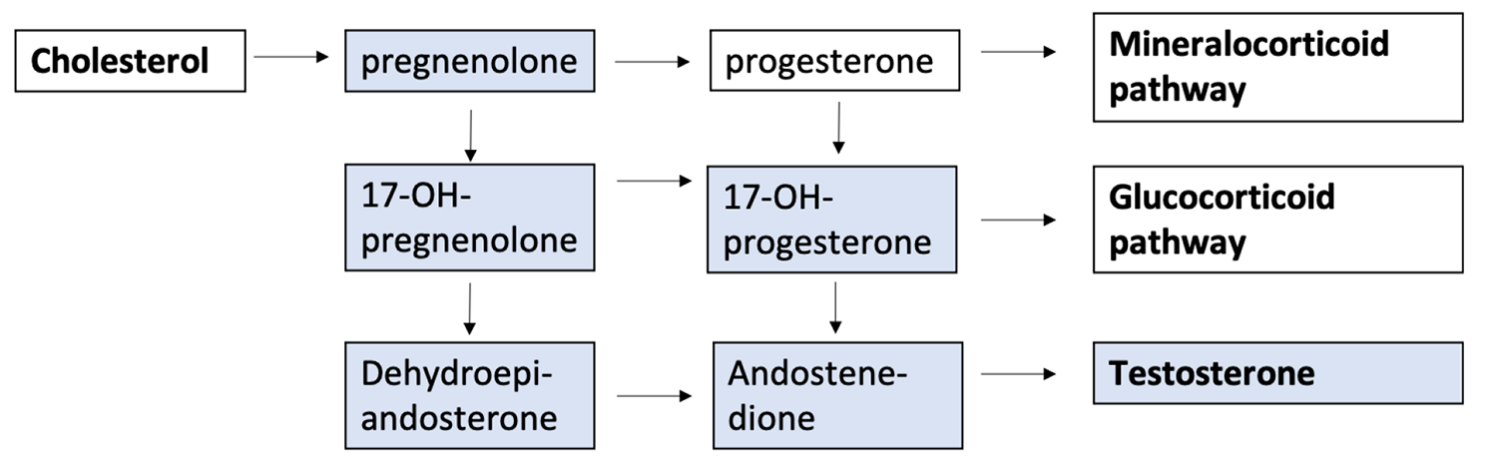
Pathways of steroid production. Steroids indicated in blue are investigated in this study. 17α-hydroxypregnenolone is indicated as 17-OH-pregnenolone; 17 α-hydroxyprogesterone is indicated as 17-OH-progesterone. The concentration of the steroids in cord blood reflects the production and metabolism in the mother, the placenta and the newborn it-self

The pathway of steroid hormone production is shown in Fig. 1 and highlights the six steroids investigated in the present study. Steroids were measured by Liquid Chromatography with tandem mass spectrometry (LC-MS-MS) using an AB Sciex 5500 triple quadrupole mass spectrometer (AB Sciex, Toronto, Canada) coupled to a Shimadzu liquid chromatography system as previously published [28]. Briefly, 200 µL of cord plasma was derivatized with hydroxylamine and analyzed using 2D chromatography on a Poros purification and Onyx C18 analytical column. All inter-assay coefficients of variation were below 10%. Testosterone values (n = 19) below LOQ (< 1 ng/dL) were substituted with LOQ/2 values (0.5 ng/dL). DHT (dihydrotestosterone) was not further investigated due to 95% (n = 378) of the measurements below the LOD (< 2.5 ng/dL). Cord plasma sex hormone-binding globulin (SHBG) was only available for 133 neonates.

### Modeling of ambient air pollution exposure

We obtained address information through questionnaires, specifically collecting details about the address at the time of delivery and the duration the mother had resided at that particular address. In cases where the mother had changed her address during pregnancy, we collected the previous address along with the dates of moving. The exposure estimates were calculated, taking into consideration the address changes during the specified periods.

- Daily regional background levels (in µg/m^3^) of PM_2.5_, BC, and NO_2_ were separately interpolated for each mother’s residential address using a spatial-temporal interpolation method (kriging) [29]. In combination with a dispersion model, this method uses air pollution data from the official fixed-site monitoring network and land-cover data obtained from satellite images (CORINE land-cover dataset; http://www.eea.europa.eu/publications/COR0-landcover) [30, 31]. This model chain provides daily BC, NO_2_ and PM_2.5_ exposure values interpolated in a high-resolution receptor grid (average grids of 25 × 25 m) using data from the Belgian telemetric air quality network, point sources, and line sources. The predictive power of the model chain was evaluated by leave-one-out cross-validation that included 34 monitoring points for PM_2.5_, 44 for NO_2_, and 14 for BC. Validation statistics of the model indicated that the spatial-temporal variability was explained by 80% for PM_2.5_[32], 74% for BC[33], and 78% for NO_2_ [32]. Our air pollution interpolation model was further validated by measuring placental BC, a gestational biomarker of prenatal exposure to BC, which correlated with the modeled BC exposure for the pregnancy period (r = 0.55; p = 0.012) [34].
- In order to show the seasonal co-fluctuation of the ambient air pollutants and meteorological factors, we included daily meteorological measurements of average temperature, relative humidity, precipitation, wind speed and sunshine in the descriptive analysis. These measurements were provided by the Royal Meteorological Institute of Belgium using the data obtained at the Diepenbeek measuring station in Limburg, Belgium. This measuring station is the nearest to the recruitment hospital (∼5.7 km), and is representative of the participants who on the average (5th to 95th percentiles), lived 13.7 km (3.8 to 25.7 km) from the station.
- Average residential air exposure levels of BC, NO_2_ and PM_2.5_ were calculated over the entire pregnancy duration representing the mean pollutant concentration (in µg/m^3^) of all pregnancy days. The date of conception was estimated based on maternity records of the last menstrual period, and if not available based on the first ultrasound exam. We also calculated the mean exposure levels of the three air pollutants for each trimester of pregnancy (1–13 weeks, 14–26 weeks, and 27 weeks until delivery). To this end, based on the daily residential ambient air exposure levels, we calculated for each mother the weekly mean BC, NO_2_ and PM_2.5_ concentrations of gestational week 1 to week 40, with week 1 starting from the estimated date of conception. In case gestational age was less than 40 weeks, the exposure levels after delivery until week 40 were set to zero. The residential address changes of 24 mothers (12%), who moved during pregnancy, were taken into account when calculating exposure levels for the different gestational time windows.

### Statistics

For the analyses, we used RStudio software (R version 4.0.2). Participant characteristics are represented as the mean +/- standard deviation (SD) for continuous variables or as the frequency (percentage) for categorical variables. The distributions of the air pollutant levels and the cord blood steroid concentrations are represented by the means, standard deviations, and interquartile ranges (IQR). Scatterplots, density plots, and Pearson correlation coefficients are used to illustrate the descriptive analysis of the steroid measurements, while raindrop plots are used for the distributions of the air pollutants. We plotted seasonal fluctuation of the daily ambient air pollutants and the following meteorological factors: daily mean temperature, relative humidity, precipitation, wind speed and sunshine. The steroid data were log10 transformed to improve the normality of the distributions.

We adopted a three-step approach for our analysis. In the first step, we conducted a screening procedure using multiple regression; in the second step, we aimed to test the consistency and further investigate the time windows of the initial findings by applying a DLM model. In the last step, we investigated a possible mixture effect of all modeled ambient air pollutants.

First, for each of the six steroids, nine linear regression models were constructed, that is per air pollutant (BC, NO_2_, and PM_2.5_) three models (trimester 1, 2 and 3) resulting in 54 models in total. Each model used the mean exposure level of the air pollutant calculated for the trimester of interest so that each cord blood steroid was modeled as the dependent variable and regressed against the trimester exposure. These models were adjusted for the following *a priori* chosen variables: the two other mean trimester exposures of the same pollutant, sex of the child, birth weight, smoking during pregnancy, gestational age, age of the mother, season of delivery, and maternal education. The models were in addition adjusted for the interaction between sex and the trimester air pollution exposures of interest. Supplementary Table 1 provides an overview of the models used and the corresponding adjustments made for the analysis. The correction for the season of delivery is implemented to account for the potential influence of seasonal effects. Because of using 6 steroids in this part, we accounted for multiple testing by adjusting the significance level to a nominal level of α = 0.01, resulting in a confidence interval (CI) of 99%. We examined whether excluding birth weight and gestational age, which are potential mediators, from the models had an impact on the obtained results.

For the associations with a p-value below the nominal level of α = 0.01, we performed sensitivity analyses, which were firstly further adjusted for ethnicity, alcohol use, parity, maternal BMI, date of delivery (to account for the sample storage time), and gestational weight gain, secondly on a subset of the data existing of mothers not receiving glucocorticoids during the pregnancy, thirdly restricting the study sample to children that were born with a vaginal birth. We adjusted the models for testosterone additionally for SHBG, however, this was run on a smaller sample size, as only 133 cord blood samples were analyzed for SHBG.

Second, the significant associations between trimester exposures of pollutants and steroid outcomes were further investigated using distributed lag models (DLMs) proposed by Gasparrini et al. [35]. The estimates were expressed as changes in the steroid outcomes for the IQR increments of the air pollutant concentration at each gestational week. The DLM model took a matrix of exposure (rows were participants and columns were weeks) as input, and the associations were smoothed over the weeks. Therefore, the exposure-response and lag-response relationships are modeled simultaneously in one model *via* the construction of a cross-basis combining two basic functions corresponding to the exposure structure and the lag structure, respectively. The exposure-response relationship was assumed to be linear, and we specified a nonlinear relationship for the lag structure using natural cubic splines with 3 inner knots equally spaced along the original lag scale (week 1 to week 40). The adjustments employed in the previous regression models remained consistent. However, two modifications were made for all DLM models: (i) the model did not account for the exposures of different trimesters, as the DLM encompassed all weekly exposures, and (ii) the interaction term of sex was omitted from the analysis, as no significant interactions were observed in the regression models. To assess the potential bias arising from multicollinearity, we generated a correlation heatmap to examine the relationship between BC and PM_2.5_ exposures for each week and we compared the estimates obtained from single time point models to those obtained from the DLM.

Third, in order to assess the mixture effect of all modeled ambient air pollutants, we performed an additional analysis taking into account: (1) the non-linearity of the response-exposure association and the interaction effects between exposures and (2) the fine time resolution of the lagged exposure effects up to weeks throughout pregnancy. Therefore, we applied the Bayesian kernel machine regression distributed lag model (BKMR-DLM), where all three air pollutants (PM_2.5_, NO_2_ and BC) were addressed simultaneously in their weekly mean format from week 0 to week 37. Participants with missing exposure values were excluded, resulting in a final sample size of N = 344. The R package “regimes” (version 0.6.30) was used to fit the model. The number of iterations was set to 10,000, with the first 5000 as the burn-in iterations. Model selection regarding the kernel type and the type and degree of freedom of the smoothing function was performed based on the Akaike information criterion (AIC) and posterior mean residual standard error. All models were adjusted for child sex, birth weight, gestational age, season of delivery, maternal age, maternal education level and smoking status during pregnancy. For testosterone, the model was also adjusted for SHBG. The BKMR-DLM identifies the critical exposure windows for each exposure. The exposure-response function of each exposure, or the main effect, is estimated as a function of time-weighted exposure for one pollutant at the median values of the time-weighted exposures of all the other pollutants. Similarly, the pairwise exposure-exposure interaction effects were estimated as a function of time-weighted exposure for one pollutant at quantiles of the time-weighted exposure of another pollutant.

## Results

### Descriptive statistics

The neonates had a mean (SD) gestational age and birth weight of 39.2 (1.5) weeks and 3412 (464) g. respectively. The sample included slightly more girls (52%) than boys, and 90% were of European ethnicity. There were fewer births in the summer (16%) than in the other seasons (25–33%) and only 17 deliveries (4%) were Caesarian. The mothers’ age, pre-pregnancy BMI and gestational weight gain averaged (SD) 29.0 (4.40) years, 24.57 (4.61) kg/m², and 14.3 (5.50) kg. respectively. Most mothers (85%) did not smoke or consume alcohol during pregnancy, and 215 mothers (54%) had a high education level (Table 1).

**Table 1:** Population characteristics (n = 397)

Neonates		Mothers	
Sex (female)	207(52.1)	Age (years)	29.0 ± 4.40
Gestational age (weeks)	39.2 ± 1.5	Education	
Birth weight (g)	3412 ± 464	Low	49(12.3)
Season of birth		Middle	134(33.8)
Winter	106(26.7)	High	214(53.9)
Spring	131(33.0)	Smoking status (yes)	59(14.9)
Summer	62(15.6)	Second-hand smoking indoor* (yes)	37(9.3)
Autumn	98(24.7)	Alcohol use (no)	336(84.6)
Ethnicity (non-European)	41(10.0)	Pre-pregnancy BMI (kg/m2)	24.57 ± 4.61
Caesarian section (yes)	16(4.0)	Gestational weight gain (kg)	14.3 ± 5.50
Preterm birth (< 37 weeks)	22 (5.5)	Parity	
Birth year		1 child	211(53.1)
2010	37(9.3)	2 or more children	186(46.9)
2011	78(19.6)		
2012	79(19.9)		
2013	22(5.5)		
2014	72(18.1)		
2015	109(27.4)		

Mean ± SD or number (%).*n = 6 missing

**Table 2:** Ambient air pollution exposure at maternal home address(es) during pregnancy (A) and steroid levels in cord blood (B)

A. Pregnancy ambient exposure (µg/m^3^)	Mean ± SD	IQR	n
Black carbon			
Entire pregnancy	1.37 ± 0.39	0.39	378
Trimester 1	1.36 ± 0.41	0.58	378
Trimester 2	1.39 ± 0.44	0.55	378
Trimester 3	1.36 ± 0.41	0.56	378
PM_2.5_			
Entire pregnancy	14.03 ± 2.35	3.30	397
Trimester 1	13.49 ± 4.86	5.47	397
Trimester 2	14.01 ± 4.31	6.51	397
Trimester 3	14.66 ± 5.08	7.96	397
NO_2_			
Entire pregnancy	18.49 ± 4.34	5.29	397
Trimester 1	18.05 ± 5.63	7.58	397
Trimester 2	18.78 ± 5.78	8.17	397
Trimester 3	18.66 ± 5.79	7.96	397
B. Steroids in cord blood (units)			
17α-hydroxypregnenolone (ng/dL)	396.23 ± 243.12	269.05	397
17α-hydroxyprogesterone (ng/dL)	2727.11 ± 1254.58	1381.75	397
DHEA: dehydroepiandrosterone (ng/dL)	166.35 ± 92.63	106.05	397
Pregnenolone (ng/dL)	1734.04 ± 890.09	791.50	397
Androstenedione (ng/dL)	54.20 ± 21.27	23.65	397
Testosterone (ng/dL)	4.26 ± 3.84	3.34	397
SHBG: Sex Hormone Binding Globulin (nmol/L)	370.36 ± 150.85	280.20	133

IQR = interquartile range

**Fig. 2:**
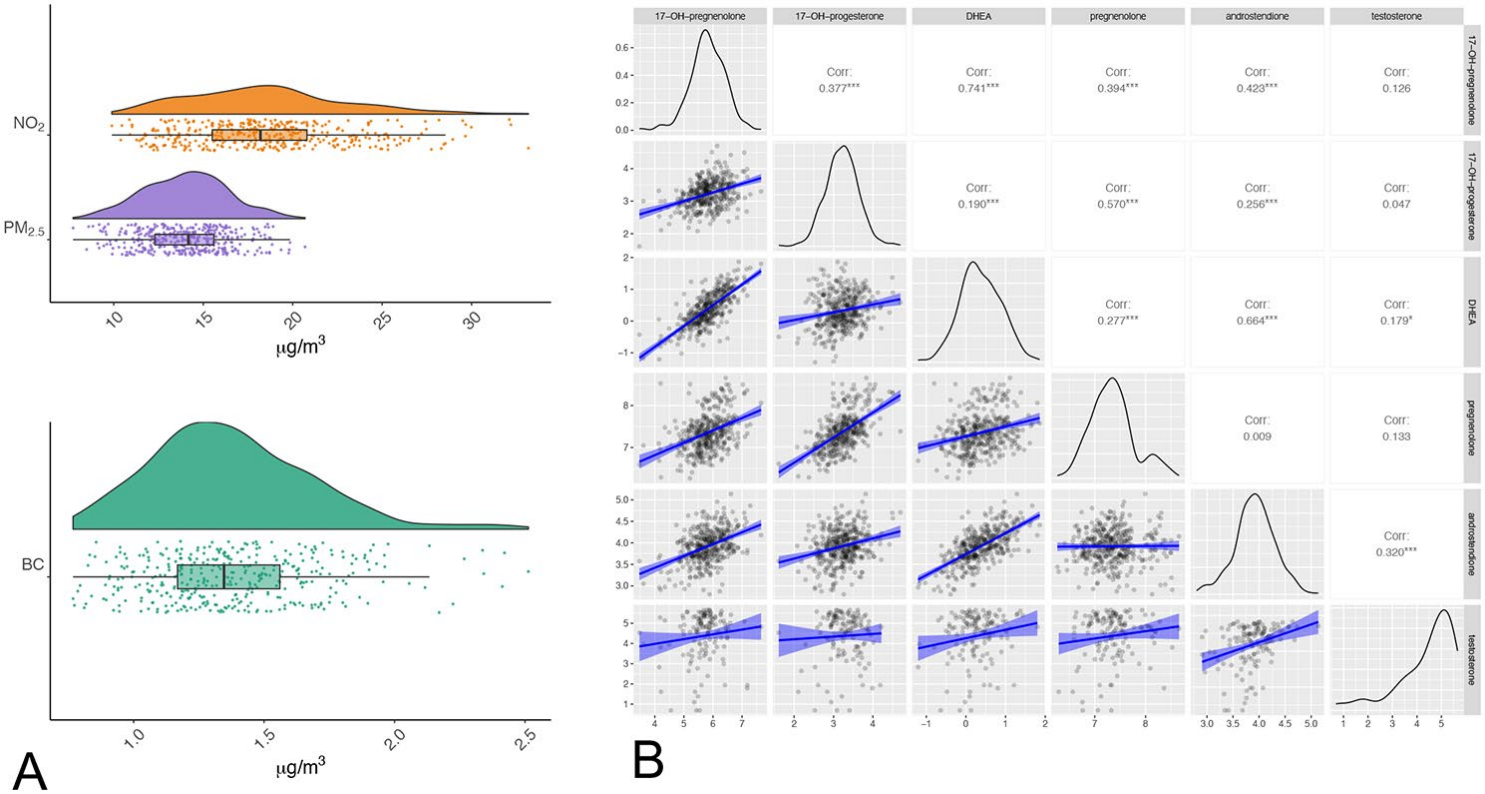
Ambient air pollution exposure at maternal home address(es) during pregnancy **(A)** and correlations between different cord blood steroid levels **(B)**. Prenatal exposure to ambient air pollutants. Raindrop plots and box-plots for and black carbon (BC, n = 378), NO_2_ and PM_2.5_ (both n = 397), all expressed as µg/m^3^. Cord blood steroids. Density plots, scatter plots, and correlation coefficients between the levels (log10 transformed) of different steroids (n = 397): 17α-hydroxypregnenolone (indicated as 17-OH-pregnenolone, ng/dL), 17α-hydroxyprogesterone (indicated as 17-OH-progesterone, ng/dL), DHEA (ng/dL), pregnenolone (ng/dL), and androstenedione (ng/dL) and testosterone (ng/dL). Corr = correlation coefficient, * indicates p < 0.05, *** indicates p < 0.0001

The exposure levels of the ambient air pollutants (mean ± SD, µg/m³) were for the whole pregnancy period 1.37 ± 0.39 for BC, 14.03 ± 2.35 for PM_2.5,_ and 18.49 ± 4.34 for NO_2_, and the exposure levels of each trimester were more or less in the same range (Table 2A, Fig. 2A). Based on the whole prenatal exposure period, the Pearson correlation coefficients between these three pregnancy air pollutant exposures were high: PM_2.5_ and BC (r = 0.68), BC and NO_2_ (r = 0.85), and PM_2.5_ and NO_2_ (r = 0.68). Exploring the correlation between the different mean trimester exposure levels of the pollutants showed that the correlations between the different mean trimester exposure levels of the pollutants were low to moderate (absolute r values ranged from 0.07 to 0.58; Supplemental Table 2). The exposure data shows seasonal fluctuations (Supplemental Fig. 1).

The mean ± SD of the log_10_-transformed concentrations of the six steroids and SHBG measured in plasma of cord blood are shown in Table 2B. Scatter plots, density plots, and correlations between these steroids are illustrated in Fig. 2B. The Pearson correlation coefficients between several steroids were high to moderate, for example, between 17α-hydroxypregnenolone and DHEA (r = 0.741), between androstenedione and DHEA (r = 0.664), and between pregnenolone and 17α-hydroxyprogesterone (r = 0.570). The correlations between other steroids, except testosterone, were weak (r = 0.190 to 0.423) or not significant.

### Association study of cord blood steroids with ambient exposure to air pollutants

**Fig. 3:**
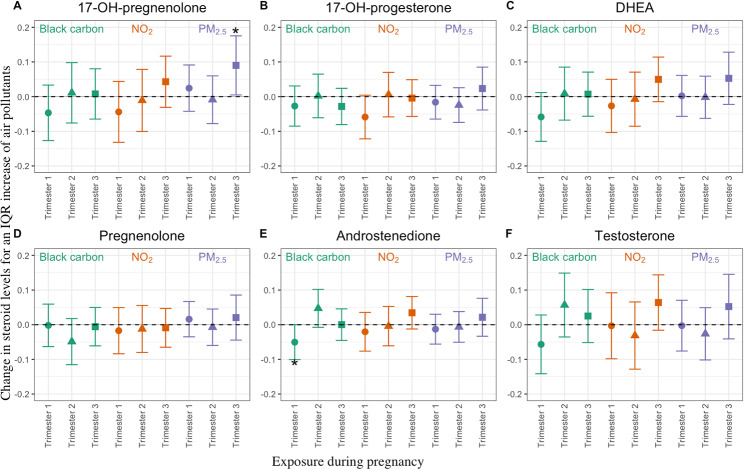
Modeling of estimated changes in cord blood steroids levels for IQR increments of trimester-specific ambient air pollution exposure at maternal home address(es) during pregnancy. Estimated changes in cord blood steroid levels for trimester-specific interquartile range (IQR) increments in black carbon, NO_2_, or PM_2.5_ exposure (in µg/m^3^) during pregnancy: **(A)** 17α-hydroxypregnenolone (indicated as 17-OH-pregnenolone), **(B)** 17α-hydroxyprogesterone (indicated as 17-OH-progesterone), **(C)** DHEA, **(D)** pregnenolone, **(E)** androstenedione, and **(F)** testosterone. The models are adjusted for the other two trimester-specific mean air pollutant exposures, sex of the child, birth weight, smoking during pregnancy, gestational age, age of the mother, the season of delivery, maternal education and interaction between sex and exposure for the trimester under study; n = 397 for PM_2.5_ and NO_2_, and n = 378 for BC. All steroid levels are expressed in ng/dL. Asterisks indicate significant estimates of the modeled associations taking into account multiple testing (CI 0.99%)

We explored the 54 association models of the cord blood steroids with the ambient exposure to each air pollutant for each of the three gestational trimesters (Fig. 3; Supplemental Table 3). Two significant associations were found between air pollutants and steroids: (i) an IQR increment of 7.96 µg/m^3^ in ambient PM_2.5_ exposure during trimester 3 was associated with an increase of 23.01% (99% confidence interval: 3.26–46.54%) in cord blood levels of 17α-hydroxypregnenolone, and (ii) an IQR increment of 0.58 µg/m³ in the ambient BC exposure during trimester 1 was associated with a decrease of 11.00% (99% CI: -19.86 to -0.012%) in cord blood levels of androstenedione. However, most of the evaluated associations were not significant. All models were adjusted for the two other trimester mean exposures of the same pollutant, sex of the child, birth weight, smoking during pregnancy, gestational age, age of the mother, the season of delivery, maternal education, and the interaction between sex and the exposure of interest. All models showed a p-value above 0.05 for the interaction term between the sex of the child and the exposure (Supplemental Table 3). We conducted an investigation to assess the potential bias arising from multicollinearity. Firstly, we generated a correlation heatmap for each week of exposure for BC and PM_2.5_, revealing a moderate correlation. The highest correlation observed for BC was 0.66, while for PM_2.5_, it was 0.62 (Supplemental Fig. 2). Secondly, we compared the estimates obtained from single time point models to those obtained from the DLM for the association between 17-OH-pregnenolone and PM_2.5_, as well as the association between androstenedione and BC. Notably, we did not find substantial inconsistencies between the two types of analyses. No significant impact was observed after excluding birth weight and gestational age, which are potential mediators, from the models on the obtained results (Supplemental Table 4).

We conducted similar associations for the three exposures using full pregnancy mean exposure which did not reveal an additional significant association, see Supplemental Table 5. In a sensitivity analysis, additional adjustments for ethnicity, alcohol use, parity, maternal BMI, date of delivery and weight gain did not mitigate the observed significance of the two association models (Table 3). Excluding 2 participants that received glucocorticoids during the pregnancy did not mitigate the observed significance of the two models. However, this restriction still showed a borderline association, with a p-value of 0.078, between PM_2.5_ exposure during trimester 3 and cord blood levels of 17α-hydroxypregnenolone. The results remained unchanged after limiting the study sample to children who were born vaginally (Table 3). Additionally adjusting the regression models for trimester exposures of testosterone for SHBG (n = 133) did not reveal significant associations (Supplemental Table 6).

**Table 3:** Sensitivity analyses for additional variables and for children born via a vaginal birth

Association model	Beta estimate	Standard error	p-value	n
a: 17α-hydroxypregnenolone ~ trimester 3 PM_2.5_ exposure	0.009	0.004	0.008	397
a: Androstenedione ~ trimester 1 BC exposure	-0.087	0.030	0.004	378
b: 17α-hydroxypregnenolone ~ trimester 3 PM_2.5_ exposure	0.019	0.003	0.001	381
b: Androstenedione ~ trimester 1 BC exposure	-0.093	0.028	0.001	362

The models for the two sensitivity analyses are adjusted for the following covariates: two other trimester-specific main air pollutant exposures, sex of the child, birth weight, smoking during pregnancy, gestational age, age of the mother, the season of delivery, and maternal education; and (a) the models were additionally adjusted for ethnicity, alcohol use, parity, maternal BMI, date of delivery, and gestational weight gain, (b) the models were restricted to children born via a vaginal birth

### Sensitive pregnancy windows for cord blood steroids in relation to ambient air pollutant exposure

**Fig. 4:**
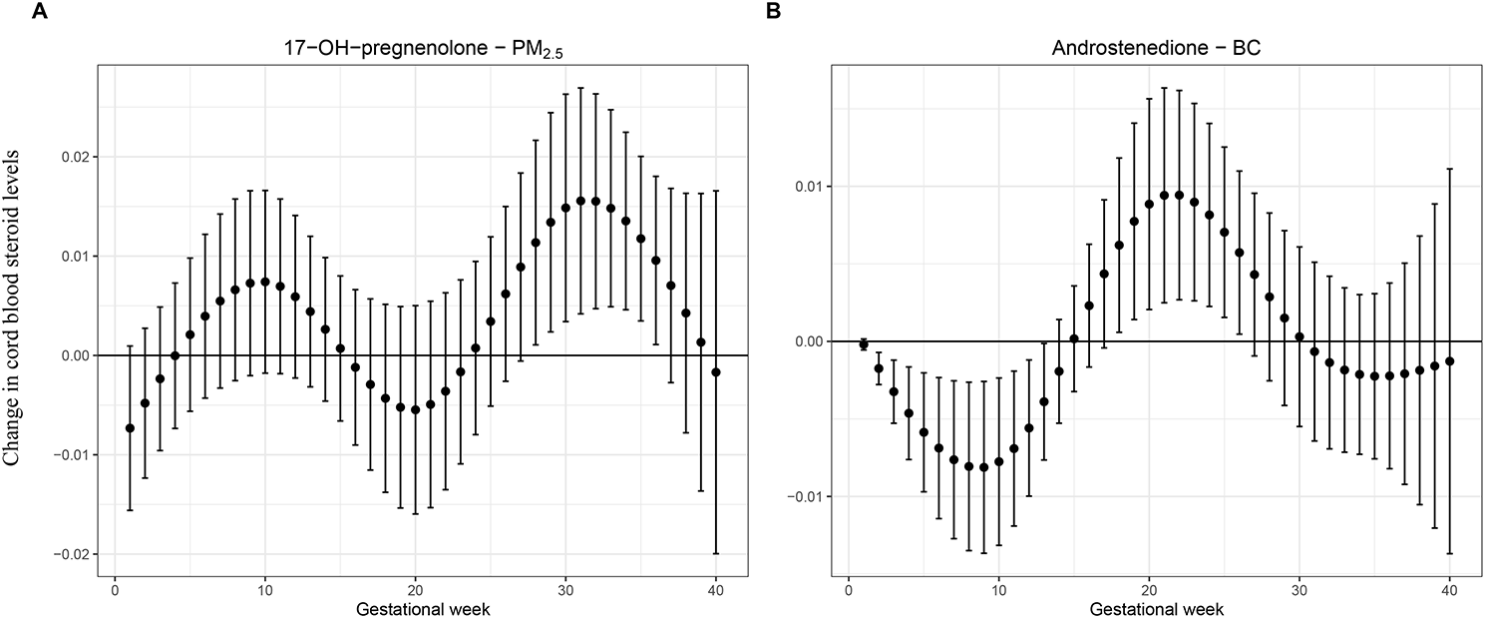
DLM models for cord blood changes of 17α-OH-pregnenolone and androstenedione with weekly mean exposures of respectively PM_2.5_ or black carbon during pregnancy. Distributed lag models to estimate the week-specific associations of the weekly IQR (interquartile range) increments of gestational exposure to the air pollutants, PM_2.5_ (n = 397) and black carbon (BC n = 378), with cord blood levels of respectively **(A)** 17α-hydroxypregnenolone, indicated as 17-OH-pregnenolone, and **(B)** androstenedione. Whiskers around the point estimates represent the confidence intervals (CI 0.95) of the week-specific estimates. Models are adjusted for sex of the child, birth weight, smoking during pregnancy, gestational age, age of the mother, the season of delivery, and maternal education

The two previously shown significant association models of cord blood 17α-hydroxypregnenolone and androstenedione with respectively ambient maternal PM_2.5_ (trimester 3) and BC (trimester 1) exposure were further studied with DLM statistics. We used DLM models to determine the estimates of the week-specific associations with the corresponding pollutant for both steroids while adjusting for lagged exposure values. This procedure allowed the identification of vulnerable gestational exposure windows to air pollutants (Fig. 4); the plots show positive and negative associations for different time windows. Cord blood 17α-hydroxypregnenolone was significantly positively associated with PM_2.5_ exposure during weeks 28–36 of pregnancy, while cord blood androstenedione was inversely related to BC exposure during gestational weeks 2–13 and positively during weeks 18–26.

### Mixture effect and critical exposure windows of PM_2.5_, NO_2_ and BC on cord blood steroids

The BKMR-DLM model selection results are shown in Supplementary Tables 7 and 8. The Gaussian kernel was selected because of the lower posterior mean residuals standard deviation. For the comparison of smoothing methods, different smoothing methods or the tuning parameters did not give substantial difference on the model goodness-of-fit. Therefore, in order to be consistent with what has been used in the DLNM, we used natural cubic splines with df = 5 as the smoothing function in the BKMR-DLM.

The critical exposure windows for each exposure are shown in Supplemental Tables 9, which was identified by testing whether the weight at time t, w(t), for an exposure is significantly different from zero. Various critical exposure windows were found for NO_2_ in 17-OH-pregnenolone, pregnenolone, androstenedione and testosterone, and for BC in pregnenolone, and testosterone. No significant main effects were identified. Interaction effects were detected between NO_2_ and BC for all steroids, and between NO_2_ and PM_2.5_ for 7-OH-Pregnenolone and DHEA (Supplemental Fig. 3). At a higher level of NO_2_ exposure, there was a more pronounced positive effect of BC exposure on 17-OH-progesterone, DHEA, pregnenolone and testosterone, compared to lower levels of NO_2_. For androstenedione, at lower NO_2_ levels the inverse effect of BC was more pronounced and at higher levels of NO_2_, there was no effect of BC. The positive effect of NO_2_ on 17-OH-pregnenolone was more pronounced at higher PM_2.5_ levels, and the positive effect of PM_2.5_ on DHEA was more pronounced at higher NO_2_ levels. No interaction effects were detected between BC and PM_2.5_.

## Discussion

The present study in neonates of the ENVIR*ON*AGE birth cohort examines ambient air pollutant exposure during pregnancy and its impact on cord blood steroids. The findings of our study suggest that the cord blood levels of 17α-hydroxypregnenolone were positively associated with third-trimester prenatal ambient exposure to PM_2.5_ and that the cord blood androstenedione levels were negatively associated with prenatal BC exposure. Nonetheless, most of the examined associations were not significant. For 17α-hydroxypregnenolone, using a DLM model, we identified a time window between 28 and 36 weeks showing a positive association with prenatal exposure to PM_2.5_. For androstenedione, the sensitive time windows were in the early (weeks 2–13, negative association) and mid-pregnancy (weeks 18–26, positive association) periods for the exposure to BC. It is of note that the cord blood concentrations of 17α-hydroxypregnenolone and androstenedione in our study are in accordance with several other studies, including a German and Czech study regarding 17α-hydroxypregnenolone [36, 37] and a Japanese and French study regarding androstenedione [5, 38]. Moreover, the cord blood concentrations of the other hormones also exhibited similar levels as previously reported by other studies [5, 37, 39, 40].

The two cord blood steroids, which were affected by air pollutant exposures, play a role in the hormonal management of the developing human organism. 17α-hydroxypregnenolone, a precursor of DHEA [41], is a neurosteroid that stimulates locomotor behavior [42]. Androstenedione is an intermediate in the biosynthesis of estrone and testosterone from DHEA and has weak androgenic activity [43]. The fetal adrenal cortex is the main site of the production of 17α-hydroxypregnenolone and androstenedione, which are converted into androgens or estrogens with the help of placental enzymes [44]. The tight coordination and regulation of these two steroid hormones provide a special reproductive endocrine environment for the fetus. We did not observe a significant association effect between air pollutants and cord blood testosterone, for which fetal DHEA is an important precursor *in utero* [44]. DHEA is also a precursor of estrogens and cortisol, for which we do not have information in our study. Further investigation is needed to unravel the role of cord blood DHEA and its downstream metabolites during pregnancy.

To our knowledge, this is the first study to address the association of prenatal exposure to air pollutants and steroid hormones in human cord blood. An experimental study showed that increased exposure to ultrafine particles of air pollution led to lower testosterone levels in male mice showing male-specific responses. The applied exposure time window was equivalent to the human third trimester in terms of brain development [26]. Our study did not show reduced levels of cord blood testosterone in relation to pregnancy air pollution exposure, however for cord blood androstenedione and 17α-hydroxypregnenolone, precursors of testosterone, significant associations were found with the air pollutants BC and PM_2.5_, respectively. A study by Zhao et al., albeit in a different study population, also did not observe a significant link between children’s testosterone blood levels and exposure to modeled annual air pollutants [16]. Considering the following three observations, — (i) the previously mentioned experimental mice study, showing in addition to the testosterone findings also alterations in social novelty preference in males, suggests that an altered steroid hormone milieu might contribute to this neurobehavioral effect [26], (ii) epidemiological studies also found sex-specific effects including neurodevelopmental outcomes associated with prenatal exposure to air pollutants [21, 23], and (iii) the exposure-related altered levels of two cord blood steroids shown in our study, — prompted us to speculate that neurological consequences of air pollution exposure might, among others, also involve changes in the metabolic levels of steroids. Circulating steroid hormones such as 17α-hydroxypregnenolone, a neuro-active steroid, modulate brain excitability by interaction with neuronal membrane receptors and ion channels primarily via GABA-A receptors [45–47]. This might be an underlying mechanism for neurodevelopmental alterations associated with early-life exposure to hazardous air pollution. Note that our previous DNA methylation study suggested a methylation enrichment in the GABAergic synapse pathway associated with early-life exposure to particulate matter [48].

Our modeled statistical analysis identified the first trimester of pregnancy as a sensitive period for BC exposure affecting cord blood androstenedione levels negatively (Fig. 3). The subsequent DLM models not only confirmed this finding but in addition revealed a positive association between both variables for the mid-pregnancy time window (Fig. 4). The reason why in the first analyses (Fig. 3) the second trimester was not identified as a significant gestational time window is most likely due to the different approaches of multiple testing. In the first modeled analyses, a more stringent significance level (α = 0.01) was applied for correlated outcomes involving six cord blood steroids. Those associations identified as significant were then subjected to DLM modeling with a nominal significance level of α = 0.05 to investigate for each gestational week the associations between cord blood steroids and exposure to air pollutants. In the human placenta, the enzyme 3β-hydroxysteroid dehydrogenase, encoded by the HSD3B1 gene, converts translocated DHEA into androstenedione, while 17α-hydroxypregnenolone is produced and metabolized in the fetus [44]. The inverse association between cord blood androstenedione and BC exposure shown for the first-trimester pregnancy may be related to altered functioning of the placenta and/or its HSD3B1 enzyme activity as a consequence of early gestational exposure to air pollution [41]. Further studies are needed to unravel the mechanism of action for such effects.

BKMR-DLM suggested exposure windows that were different from the DLNM. The results for BC were relatively consistent, while the significant exposure window detected for PM_2.5_ by DLM was not detected by BKMR-DLM, and BKMR-DLM identified exposure windows for NO_2_ which were not shown by DLM. When a mixture of pollutants was included in the model, the estimated effect of each pollutant was conditional on other pollutants, which might explain the discrepancy between the two models. However, the effect direction of exposures at the exposure windows could not be estimated in BKMR-DLM and only null main effects were observed; it is therefore not feasible to see if the direction of BC effect was consistent or not within the same gestational weeks. An important added value of the BKMR-DLM was to identify interaction effects between pollutants, which has been suggested especially for NO_2_. NO_2_ is an air pollutant that is primarily traffic-related and a higher NO_2_ level at residence might be an indicator of living closer to major traffic roads or living in more urbanized regions. At a higher NO_2_ level, exposures to other air pollutants were shown to have stimulated the level of steroids in cord blood, although whether it is a protective mechanism or dysregulation remains further investigation.

An underlying mechanism of negative health effects of exposure to airborne particles may be oxidative stress and inflammation [49–51]. In addition, inhaling polluted air may be a possible route of exposure to endocrine-disrupting compounds (EDCs) [6] Outdoor air can contain a range of EDCs originating from agriculture, industrial activities, waste incineration or combustion fumes of petrol and diesel [7].

The strengths of our study are the following: i) we estimated ambient air pollution exposure for BC, NO_2_, and PM_2.5_ using a well-validated model with high predictive accuracy. Further, for each of the three air pollutants, we used the weekly mean exposures corresponding to each gestational week rather than mean the exposures of each pregnancy trimester and employed them in DLMs that enabled us to identify sensitive time windows per week of gestation; ii), we applied correction for multiple testing; and iii) an important strength is the use of a highly specific multiplex mass spectrometry method for determining the cord blood steroid concentrations as compared to less specific and/or less sensitive immunoassays.

There are also limitations of our study. As the steroid hormone levels were measured in cord blood, they may be less adequate to represent the infant endocrine system because at the end of gestation, the hypothalamic-pituitary-gonadal (HPG) axis is inhibited due to a negative feedback controlled by placental hormones [52]. Following birth, the HPG axis undergoes reactivation until the infant reaches six months of age and at around three months, there is a notable surge in sex steroid concentrations [53]. There is, however, a growing number of evidence that the analysis of steroids in cord blood may be linked to endocrine diseases during the lifespan [54]. We do not have information on the downstream metabolites of the steroids. We lack information on the chemical composition of particulate matter, which could provide opportunities to further explain possible endocrine disturbing actions of the particles. In the (constrained) DLM model, the smoothing of association estimates at neighboring time points might have stretched the shape of the curve to suggest significance that is not necessarily true. In addition, collinearity might also be an explanation as suggested by Basagana and Barrera-Gómez [55], although the intercorrelation between exposure levels of different gestational weeks is only moderate in our data (Supplementary Fig. 2). While our exposure data does exhibit variation, it is important to note that some of this variation is attributed to seasonal fluctuations and that the study population’s air exposure levels are below the annual average threshold of 25 µg/m^3^ for PM_2.5_ air pollution set by the European Union, and above the World Health Organization’s (WHO) recommended guideline value of 5 µg/m^3^ for PM_2.5_ (annual average) and 10 µg/m^3^ for NO_2_ [56, 57]. We used the maternal home address as a proxy for estimating personal exposure to air pollutants, which does not include all locations mothers have been exposed to ambient air pollution. It represents the primary location where individuals are likely to experience prolonged and repeated exposure and is, therefore, relevant for estimating the exposure to ambient air pollutants. We recognize the absence of certain factors that could potentially confound the associations between ambient air pollution and steroids in cord blood. These factors include road traffic noise and other endocrine-disrupting compounds that may be associated with the place of residence and the influence of collinearity. In addition, maternal and paternal exposure to ambient air pollution preceding pregnancy, although not the focus of this study, could have an impact on conception, maternal and placental steroids, pregnancy outcomes, and the observed associations between steroids and ambient air pollution exposure, highlighting the need for future research to explore a potential source of bias.

In conclusion, our study revealed potential links between neonate cord blood steroid levels and ambient pregnancy exposure to air pollutants. More specifically, sensitive gestational time windows of air pollutant exposure were associated with changes in cord blood steroid levels such as 17α-hydroxypregnenolone for third-trimester PM_2.5_ exposure and androstenedione for first-trimester and mid-pregnancy BC exposure. We identified interaction effects between pollutants, which were suggested especially for NO_2_. Further studies should investigate potential mechanisms of action and possible later-in-life adverse consequences of hormonal disturbances due to air pollution exposure.

### Electronic supplementary material

Below is the link to the electronic supplementary material.

Supplementary Material 1

## Data Availability

Code and data relevant to the analyses are available upon request to the authors.

## Abbreviations

PM_2.5_: Particles with aerodynamic diameter ≤ 2.5 μm
NO_2_: Nitrogen dioxide
BC: Black carbon
DLM: Distributed lag models
IQR: Interquartile range
EDCs: Endocrine-disrupting compounds
DHEA: Dehydro-epiandrosterone
17α-hydroxyprogesterone: 17-OH-progesterone
17α-hydroxypregnenolone: 17-OH-pregnenolone
ENVIR*ON*AGE: ENVIRONmental influence *ON* AGEing in early life
LC-MS-MS: Liquid Chromatography with tandem Mass Spectrometry
BKMR-DLM: Bayesian kernel machine regression distributed lag model
