# Supplementary material for: The association between newborn cord blood steroids and ambient prenatal exposure to air pollution: findings from the ENVIRONAGE birth cohort
Source: Environ Health. 2023 Sep 7;22:63. doi: 10.1186/s12940-023-01010-w (PMC10483875; doi:10.1186/s12940-023-01010-w)
Supplement: Supplementary file 1 — Supplementary Material 1 [file 12940_2023_1010_MOESM1_ESM.docx]

**Supplemental Table 1: Correlations between the gestational trimesters of ambient exposure to black carbon (BC), particulate matter (PM2.5), and nitrogendioxide (NO2).**

|  | trimester 1 BC | trimester 2 BC | trimester 3 BC |
| --- | --- | --- | --- |
| trimester 1 BC | 1 | 0.30 | 0.07 |
| trimester 2 BC | 0.30 | 1 | 0.52 |
| trimester 3 BC | 0.07 | 0.52 | 1 |
|  |  |  |  |
|  | trimester 1 PM2.5 | trimester 2 PM2.5 | trimester 3 PM2.5 |
| trimester 1 PM_2.5_ | 1 | -0.08 | -0.55 |
| trimester 2 PM_2.5_ | -0.08 | 1 | 0.30 |
| trimester 3 PM_2.5_ | -0.55 | 0.30 | 1 |
|  |  |  |  |
|  | trimester 1 NO2 | trimester 2 NO2 | trimester 3 NO2 |
| trimester 1 NO_2_ | 1 | 0.42 | 0.08 |
| trimester 2 NO_2_ | 0.42 | 1 | 0.58 |
| trimester 3 NO_2_ | 0.08 | 0.58 | 1 |

PM_2.5_ and NO2, n=397; BC, n=378.

**Supplemental Table 2: Associations between cord blood steroid levels and trimester-specific air exposures to black carbon (BC), nitrogen dioxide (NO_2_), and particulate matter (PM_2.5_).**

| **Number of observations** | **Steroid** | **Time point** | **Exposure** | **Beta-estimate** | **standard error** | **raw p value** | **p value for interaction sex** |
| --- | --- | --- | --- | --- | --- | --- | --- |
| 378 | 17 α-OH pregnenolone | Trimester 1 | BC | -0.0811 | 0.0536 | 0.1315 | 0.2069 |
| 378 | 17 α-OH pregnenolone | Trimester 2 | BC | 0.0200 | 0.0612 | 0.7437 | 0.3038 |
| 378 | 17 α-OH pregnenolone | Trimester 3 | BC | 0.0139 | 0.0498 | 0.7804 | 0.6474 |
| 397 | 17 α-OH pregnenolone | Trimester 1 | NO_2_ | -0.0058 | 0.0045 | 0.1952 | 0.0864 |
| 397 | 17 α-OH pregnenolone | Trimester 2 | NO_2_ | -0.0013 | 0.0043 | 0.7528 | 0.2748 |
| 397 | 17 α-OH pregnenolone | Trimester 3 | NO_2_ | 0.0054 | 0.0036 | 0.1327 | 0.6191 |
| 397 | 17 α-OH pregnenolone | Trimester 1 | PM_2.5_ | 0.0045 | 0.0047 | 0.3432 | 0.1114 |
| 397 | 17 α-OH pregnenolone | Trimester 2 | PM_2.5_ | -0.0014 | 0.0041 | 0.7380 | 0.5605 |
| 397 | 17 α-OH pregnenolone | Trimester 3 | PM_2.5_ | 0.0113 | 0.0041 | 0.0065* | 0.9365 |
| 378 | 17 α-OH progesterone | Trimester 1 | BC | -0.0470 | 0.0388 | 0.2263 | 0.2610 |
| 378 | 17 α-OH progesterone | Trimester 2 | BC | 0.0038 | 0.0442 | 0.9316 | 0.6230 |
| 378 | 17 α-OH progesterone | Trimester 3 | BC | -0.0504 | 0.0360 | 0.1626 | 0.9082 |
| 397 | 17 α-OH progesterone | Trimester 1 | NO_2_ | -0.0077 | 0.0032 | 0.0160 | 0.0897 |
| 397 | 17 α-OH progesterone | Trimester 2 | NO_2_ | 0.0007 | 0.0030 | 0.8158 | 0.2324 |
| 397 | 17 α-OH progesterone | Trimester 3 | NO_2_ | -0.0005 | 0.0026 | 0.8396 | 0.8722 |
| 397 | 17 α-OH progesterone | Trimester 1 | PM_2.5_ | -0.0029 | 0.0034 | 0.3923 | 0.3974 |
| 397 | 17 α-OH progesterone | Trimester 2 | PM_2.5_ | -0.0038 | 0.0030 | 0.2094 | 0.4488 |
| 397 | 17 α-OH progesterone | Trimester 3 | PM_2.5_ | 0.0029 | 0.0030 | 0.3272 | 0.4347 |
| 378 | DHEA | Trimester 1 | BC | -0.1017 | 0.0471 | 0.0317 | 0.2152 |
| 378 | DHEA | Trimester 2 | BC | 0.0159 | 0.0537 | 0.7678 | 0.2619 |
| 378 | DHEA | Trimester 3 | BC | 0.0128 | 0.0437 | 0.7696 | 0.2381 |
| 397 | DHEA | Trimester 1 | NO_2_ | -0.0035 | 0.0039 | 0.3687 | 0.2563 |
| 397 | DHEA | Trimester 2 | NO_2_ | -0.0009 | 0.0037 | 0.8083 | 0.6029 |
| 397 | DHEA | Trimester 3 | NO_2_ | 0.0063 | 0.0031 | 0.0459 | 0.7266 |
| 397 | DHEA | Trimester 1 | PM_2.5_ | 0.0004 | 0.0042 | 0.9242 | 0.1665 |
| 397 | DHEA | Trimester 2 | PM_2.5_ | -0.0003 | 0.0036 | 0.9385 | 0.8998 |
| 397 | DHEA | Trimester 3 | PM_2.5_ | 0.0066 | 0.0037 | 0.0698 | 0.9662 |
| 378 | pregnenolone | Trimester 1 | BC | -0.0032 | 0.0411 | 0.9387 | 0.5287 |
| 378 | pregnenolone | Trimester 2 | BC | -0.0888 | 0.0468 | 0.0583 | 0.1925 |
| 378 | pregnenolone | Trimester 3 | BC | -0.0100 | 0.0381 | 0.7932 | 0.4016 |
| 397 | pregnenolone | Trimester 1 | NO_2_ | -0.0023 | 0.0034 | 0.5019 | 0.4587 |
| 397 | pregnenolone | Trimester 2 | NO_2_ | -0.0015 | 0.0032 | 0.6417 | 0.4312 |
| 397 | pregnenolone | Trimester 3 | NO_2_ | -0.0011 | 0.0027 | 0.6819 | 0.5706 |
| 397 | pregnenolone | Trimester 1 | PM_2.5_ | 0.0029 | 0.0036 | 0.4192 | 0.4119 |
| 397 | pregnenolone | Trimester 2 | PM_2.5_ | -0.0011 | 0.0031 | 0.7185 | 0.6669 |
| 397 | pregnenolone | Trimester 3 | PM_2.5_ | 0.0026 | 0.0032 | 0.4097 | 0.7891 |
| 378 | androstendione | Trimester 1 | BC | -0.0873 | 0.0337 | 0.0099* | 0.9685 |
| 378 | androstendione | Trimester 2 | BC | 0.0859 | 0.0384 | 0.0258 | 0.7488 |
| 378 | androstendione | Trimester 3 | BC | 0.0004 | 0.0312 | 0.9898 | 0.8138 |
| 397 | androstendione | Trimester 1 | NO_2_ | -0.0027 | 0.0028 | 0.3451 | 0.6008 |
| 397 | androstendione | Trimester 2 | NO_2_ | -0.0005 | 0.0027 | 0.8504 | 0.7883 |
| 397 | androstendione | Trimester 3 | NO2 | 0.0043 | 0.0023 | 0.0573 | 0.6228 |
| 397 | androstendione | Trimester 1 | PM_2.5_ | -0.0024 | 0.0030 | 0.4395 | 0.7843 |
| 397 | androstendione | Trimester 2 | PM_2.5_ | -0.0010 | 0.0026 | 0.7101 | 0.5724 |
| 397 | androstendione | Trimester 3 | PM_2.5_ | 0.0027 | 0.0027 | 0.3091 | 0.2730 |
| 378 | testosterone | Trimester 1 | BC | -0.0982 | 0.0568 | 0.0846 | 0.5194 |
| 378 | testosterone | Trimester 2 | BC | 0.1032 | 0.0647 | 0.1116 | 0.4262 |
| 378 | testosterone | Trimester 3 | BC | 0.0446 | 0.0526 | 0.3976 | 0.3473 |
| 397 | testosterone | Trimester 1 | NO2 | -0.0004 | 0.0048 | 0.9339 | 0.1927 |
| 397 | testosterone | Trimester 2 | NO2 | -0.0039 | 0.0046 | 0.4035 | 0.7777 |
| 397 | testosterone | Trimester 3 | NO2 | 0.0080 | 0.0039 | 0.0386 | 0.2640 |
| 397 | testosterone | Trimester 1 | PM2.5 | -0.0005 | 0.0051 | 0.9230 | 0.1145 |
| 397 | testosterone | Trimester 2 | PM2.5 | -0.0041 | 0.0045 | 0.3681 | 0.5542 |
| 397 | testosterone | Trimester 3 | PM2.5 | 0.0065 | 0.0045 | 0.1472 | 0.1469 |

**Suppl. Table 2**: Beta-estimates, standard error, and raw p-values of regression models of the cord blood steroids with BC , PM_2.5,_ or NO_2_ exposure during the different trimesters of pregnancy. Each model is adjusted for the two other trimester-specific mean exposures to air pollutants, sex of the child, birth weight, smoking during pregnancy, gestational age, age of the mother, the season of delivery, maternal education, and interaction between sex and exposure for the trimester under study. The p-values of interaction are for the interaction between the sex of the neonate and the air pollutant exposure under study for a specific trimester. Asterisks indicate significant associations taking into account multiple testing (p<0.001).

**Supplemental Table 3: Associations between cord blood testosterone levels and trimester-specific air exposures to black carbon (BC), nitrogen dioxide (NO_2_), and particulate matter (PM_2.5_), additionally adjusted for SHBG.**

| **Number of observations** | **Time point** | **Exposure** | **Beta-estimate** | **standard error** | **raw p value** | **p value for interaction sex** |
| --- | --- | --- | --- | --- | --- | --- |
| 129 | Trimester 1 | BC | -0.0985 | 0.0933 | 0.2930 | 0.5708 |
| 129 | Trimester 2 | BC | 0.0340 | 0.1095 | 0.7566 | 0.6240 |
| 129 | Trimester 3 | BC | 0.0627 | 0.0916 | 0.4948 | 0.8028 |
| 133 | Trimester 1 | NO2 | -0.0109 | 0.0070 | 0.1208 | 0.6781 |
| 133 | Trimester 2 | NO2 | -0.0068 | 0.0064 | 0.2874 | 0.6150 |
| 133 | Trimester 3 | NO2 | 0.0002 | 0.0071 | 0.9780 | 0.5040 |
| 133 | Trimester 1 | PM2.5 | -0.0140 | 0.0176 | 0.4293 | 0.9120 |
| 133 | Trimester 2 | PM2.5 | -0.0017 | 0.0105 | 0.8681 | 0.8194 |
| 133 | Trimester 3 | PM2.5 | 0.0096 | 0.0112 | 0.3952 | 0.9144 |

**Suppl. Table 2**: Beta-estimates, standard error, and raw p-values of regression models of the cord blood steroids with BC , PM_2.5,_ or NO_2_ exposure during the different trimesters of pregnancy. Each model is adjusted for the two other trimester-specific mean exposures to air pollutants, sex of the child, birth weight, smoking during pregnancy, gestational age, age of the mother, the season of delivery, maternal education, SHBG, and interaction between sex and exposure for the trimester under study. The p-values of interaction are for the interaction between the sex of the neonate and the air pollutant exposure under study for a specific trimester.
